# Supplementary material for: Exercise Promotes Pro-Apoptotic Ceramide Signaling in a Mouse Melanoma Model
Source: Cancers (Basel). 2022 Sep 2;14(17):4306. doi: 10.3390/cancers14174306 (PMC9454537; doi:10.3390/cancers14174306)
Supplement: Supplementary file 1 [file cancers-14-04306-s001.zip › Supplementary_Material_Figures S1-S5_Table S4_JL.pdf]

Supplementary Material

1 Supplementary Figures and Tables

1.1 Supplementary Figures

1.1.1 Supplementary Figure S1. Experimental schema

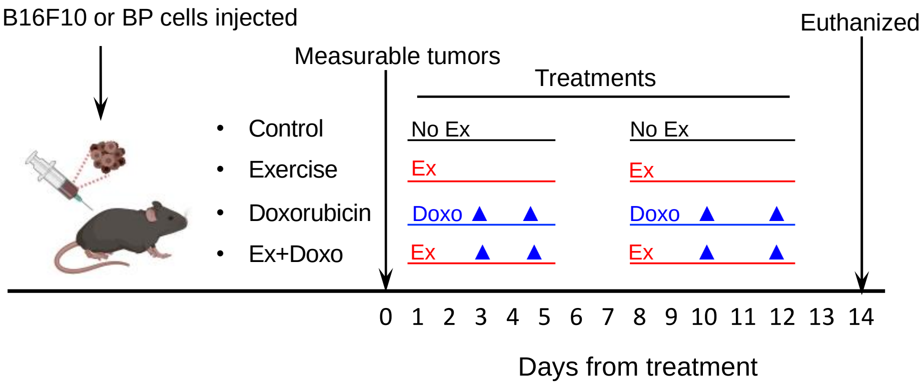

Melanoma cell lines

- B16F10: Ink4a/Arf $^{-/-}$, Braf WT, p53 WT
- BP: Braf^{V600E}/Pten$^{-/-}$, p53 WT

Treatments

- Ex (Treadmill running)
  - 12 meter/min (≈ 65% V̇O2max, a brisk walk for humans)
  - 45 min/day x 5 days
- Doxo (▲)
  - Tail vein injection
  - 2.5 mg/kg x 4 = 10 mg/kg

Supplementary Figure S1. Animal experimental schema. C57Bl/6 male mice were injected with B16F10 or BP tumor cells. When tumors were measurable, mice were divided into four groups: control (No Ex or Con), exercise (Ex), doxorubicin (Doxo), or exercise plus doxorubicin (Ex+Doxo). Ex consisted of moderate treadmill running at 12 meters/minute for 45 min for 5 days per week for 2 weeks. Doxo at a concentration of 2.5 mg/kg in 100 μL of PBS was intravenously administered four times (twice per week). Tumor volumes were measured every other day. All mice were euthanized at 14 days following the initiation of exercise in the Ex group.

1.1.2 Supplementary Figure S2

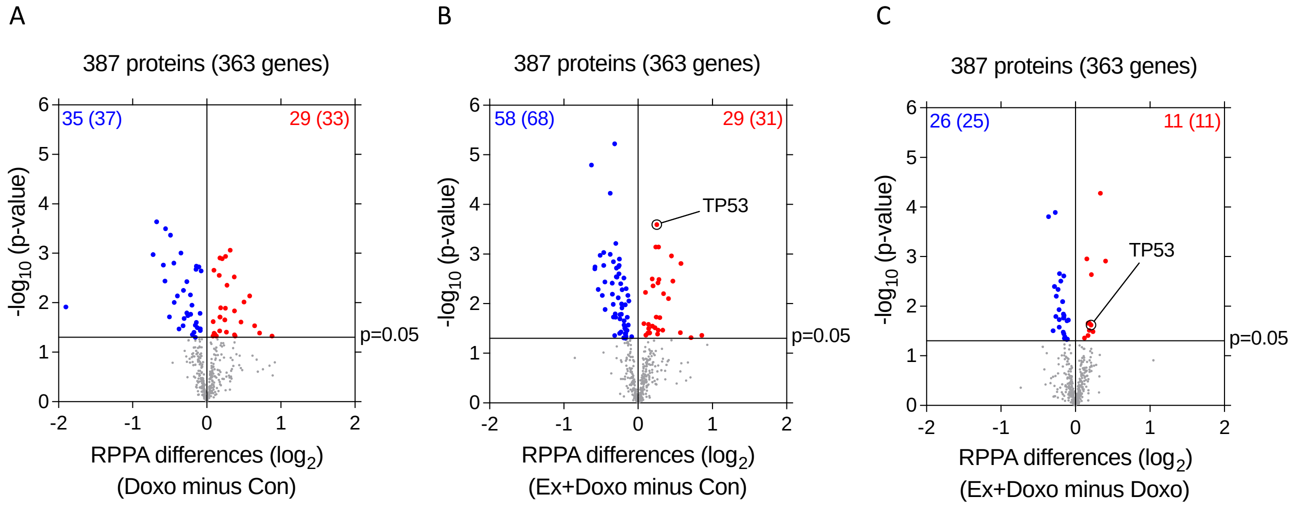

Supplemental Figure S2. Volcano plot analysis. **(A-C)** Volcano plot analyses were performed to identify significant differences of the 387 proteins evaluated in the RPPA protein set between tumors from Doxo vs Con **(A)**, Ex+Doxo vs Con **(B)**, and Ex+Doxo vs Doxo **(C)** mice. Blue and red indicate downregulated and upregulated proteins on each plot. Horizontal line is p-value set at 0.05.

1.1.2 Supplementary Figure S3

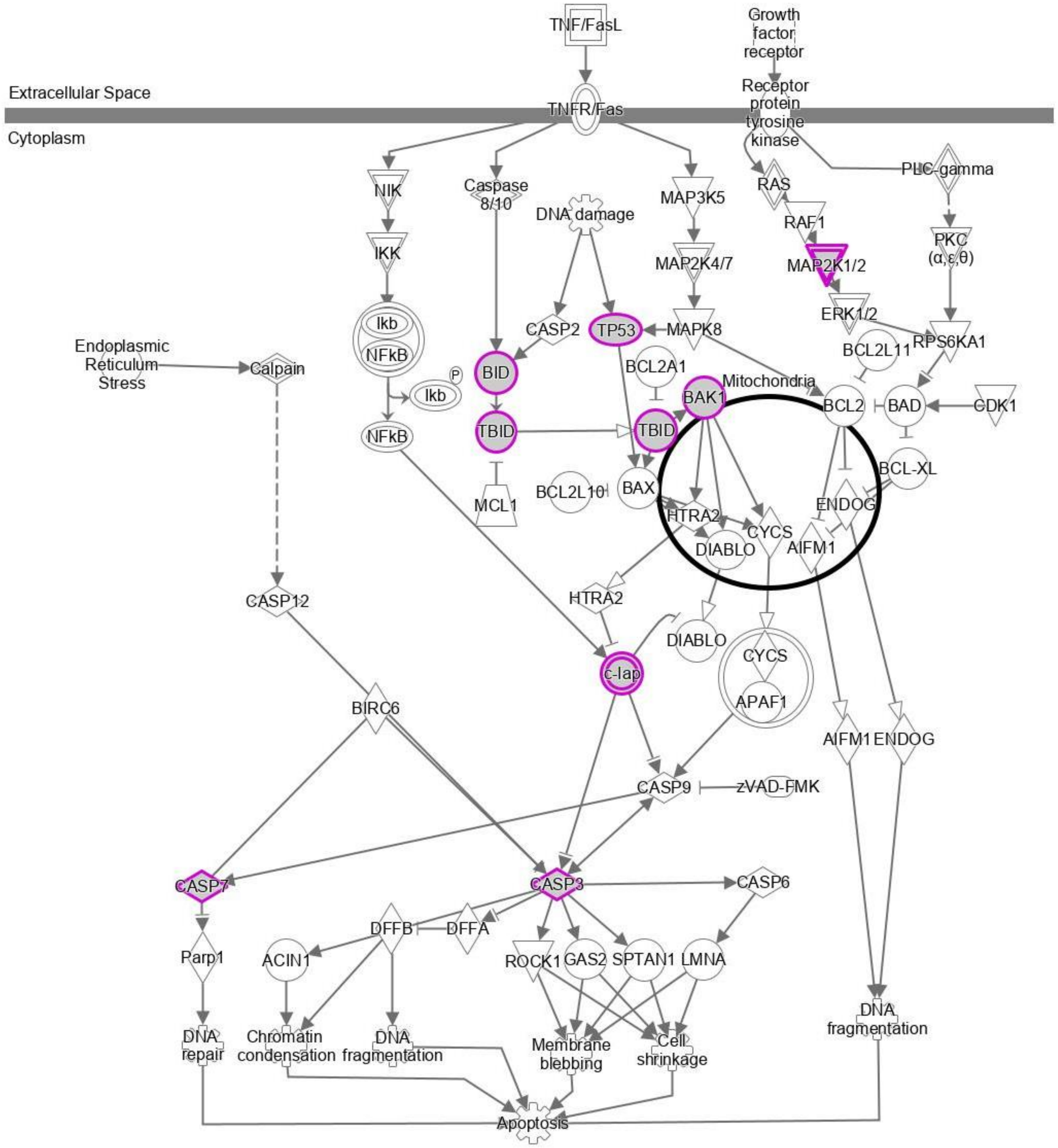

Supplementary Figure S3. Gene pathway analysis. IPA (Ingenuity Pathway Analysis) for exercise-induced canonical apoptosis signaling in B16F10 tumors. Significantly regulated proteins are denoted by a pink outline. $p<0.05$.

1.1.3 Supplementary Figure S4

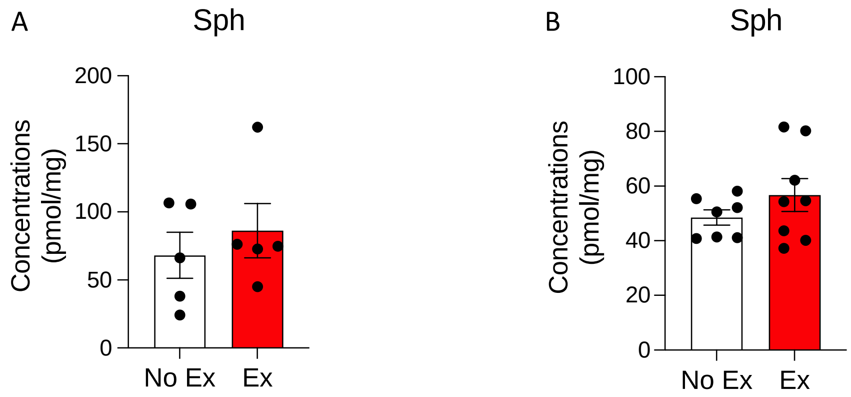

Supplementary Figure S4. No effect of exercise on sphingosine (Sph) in tumors. Sph levels in homogenates of B16F10 **(A)** and BP **(B)** tumors were analyzed by LC/MS. Absolute values of sphingosine molecule were represented as mean ± SEM in bar graph. Sph: Sphingosine.

1.1.4 Supplementary Figure S5

Figure 4C

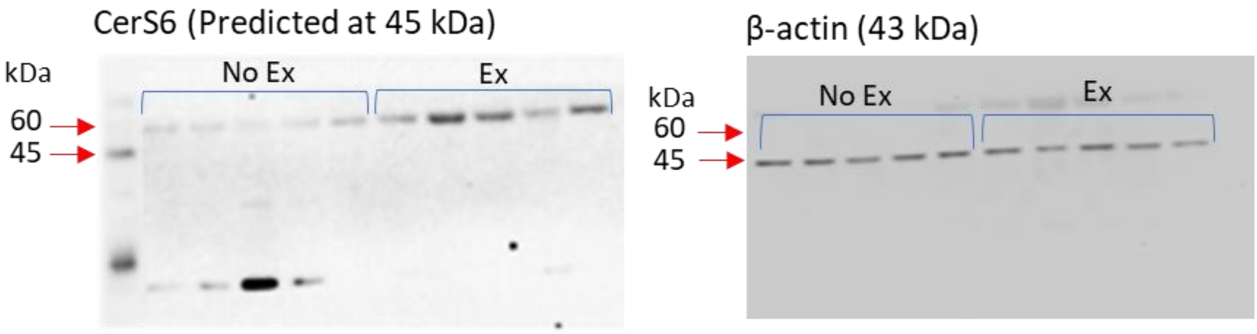

Absolute intensity

CerS6	1	2	3	4	5
No Ex	3880.669	3581.426	3821.548	4692.205	6672.376
Ex	10208.79	18843.083	14792.083	9442.669	13224.083

β-actin	1	2	3	4	5
No Ex	13970.5947	14075.686	13709.605	14641.686	15927.0023
Ex	16280.7167	15467.807	19953.5787	16079.238	9733.393

Internal normalization

CerS6/β-actir	1	2	3	4	5
No Ex	0.28	0.25	0.28	0.32	0.42
Ex	0.63	1.22	0.74	0.59	1.36

0.31	1	2	3	4	5
No Ex	0.90	0.82	0.90	1.03	1.35
Ex	2.02	3.93	2.39	1.89	4.38

Fold change to NoEx

	Aver.	SEM	
No Ex	1.00	0.09	
Ex	2.92	0.51	

Figure 4D

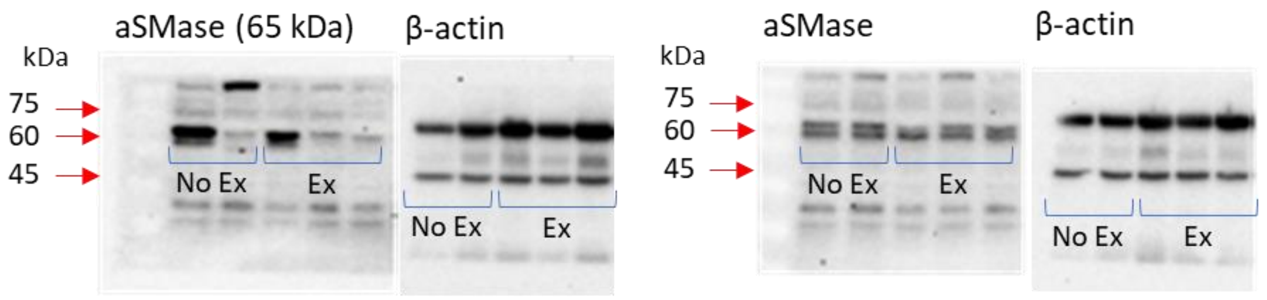

Absolute intensity

aSMase	1	2	3	4	5	6
No Ex	19724.912	6604.134		16858.205	18491.841	
Ex	12947.255	6819.719	4424.719	14190.083	15681.548	14653.205
β-actin	1	2	3	4	5	6
No Ex	10712.912	11397.841		10380.326	11513.376	
Ex	14556.376	12092.719	17045.083	14245.083	13822.79	11651.669

Internal normalization

aSMase/β-ac	1	2	3	4	5	6
No Ex	1.84	0.58		1.62	1.61	
Ex	0.89	0.56	0.26	1.00	1.13	1.26
1.41	1	2	3	4	5	6
No Ex	1.30	0.41		1.15	1.14	
Ex	0.63	0.40	0.18	0.71	0.80	0.89

Fold change to NoEx

	Aver.	SEM
No Ex	1.00	0.20
Ex	0.54	0.11

Figure 4E

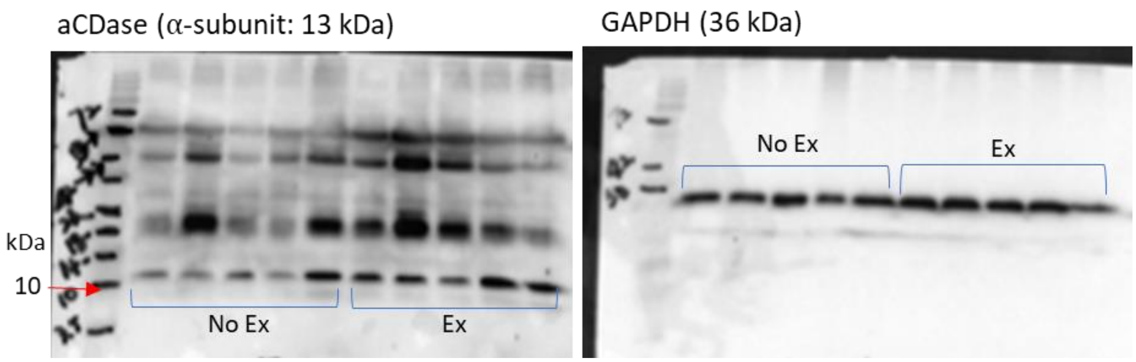

Absolute intensity					
aCDase	1	2	3	4	5
No Ex	6216.25483	7261.54773	14864.5477	7021.7193	5546.01219
Ex	11111.4264	10465.3762	7070.01219	16031.5477	12016.8406
GAPDH	1	2	3	4	5
No Ex	9755.96194	9116.25483	14788.8406	14319.8406	7916.89087
Ex	16612.3051	17275.0122	15179.4264	15667.1335	8123.37615

Internal normalization					
aCDase/GAP	1	2	3	4	5
No Ex	0.64	0.80	1.01	0.49	0.70
Ex	0.67	0.61	0.47	1.02	1.48
0.73	1	2	3	4	5
No Ex	0.88	1.10	1.38	0.68	0.96
Ex	0.92	0.83	0.64	1.41	2.04

Fold change to NoEx		
	Aver.	SEM
No Ex	1.00	0.12
Ex	1.23	0.25

Figure 4F

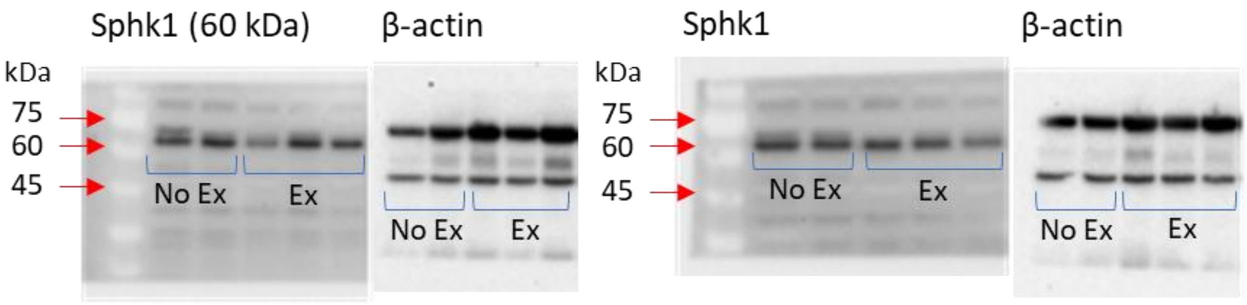

Absolute intensity						
**Sphk1**	**1**	**2**	**3**	**4**	**5**	**6**
**No Ex**	16479.962	16704.962		19816.79	17743.548	
**Ex**	10123.184	16283.134	12236.719	15584.376	13937.79	11630.669
**β-actin**	**1**	**2**	**3**	**4**	**5**	**6**
**No Ex**	10712.912	11397.841		10380.326	11513.376	
**Ex**	14556.376	12092.719	17045.083	14245.083	13822.79	11651.669

Internal normalization						
**Sphk1/β-acti**	**1**	**2**	**3**	**4**	**5**	**6**
**No Ex**	1.54	1.47		1.91	1.54	
**Ex**	0.70	1.35	0.72	1.09	1.01	1.00
**1.61**	**1**	**2**	**3**	**4**	**5**	**6**
**No Ex**	0.95	0.91		1.18	0.96	
**Ex**	0.43	0.83	0.44	0.68	0.62	0.62

Fold change to NoEx		
	**Aver.**	**SEM**
**No Ex**	1.00	0.06
**Ex**	0.61	0.08

Figure 5D

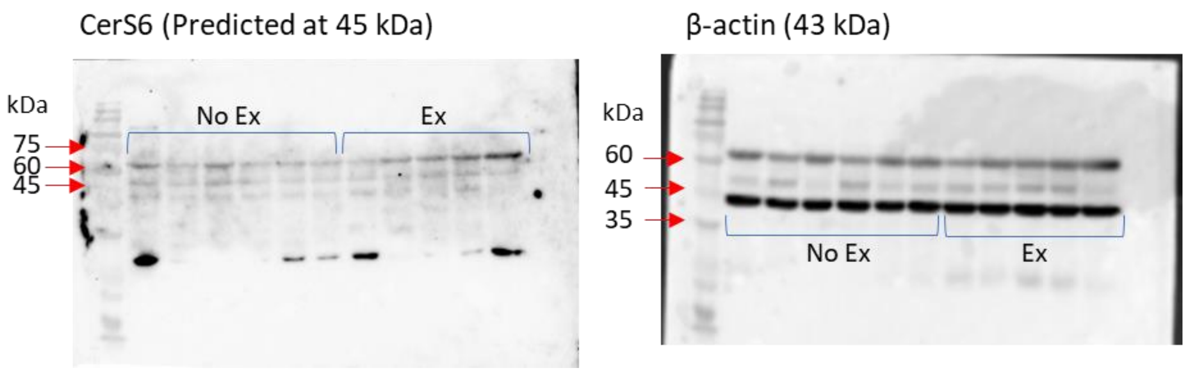

Absolute intensity						
**CerS6**	**1**	**2**	**3**	**4**	**5**	**6**
**No Ex**	10728.91	9033.255	11978.84	6757.719	10636.26	8172.79
**Ex**	13737.84	10349.13	11537.26	6429.426	13612.96	
**β-actin**	**1**	**2**	**3**	**4**	**5**	**6**
**No Ex**	15840.79	15640.72	13053.55	14815.01	13295.6	12867.84
**Ex**	13255.13	14477.01	14399.01	15165.43	13720.43	

Internal normalization						
**CerS6/β-a**	**1**	**2**	**3**	**4**	**5**	**6**
**No Ex**	0.68	0.58	0.92	0.46	0.80	0.64
**Ex**	1.04	0.71	0.80	0.42	0.99	
**0.68**	**1**	**2**	**3**	**4**	**5**	**6**
**No Ex**	1.00	0.85	1.35	0.67	1.18	0.94
**Ex**	1.53	1.06	1.18	0.63	1.46	

Fold change to control		
	**Aver.**	**SEM**
**No Ex**	1.00	0.10
**Ex**	1.17	0.16

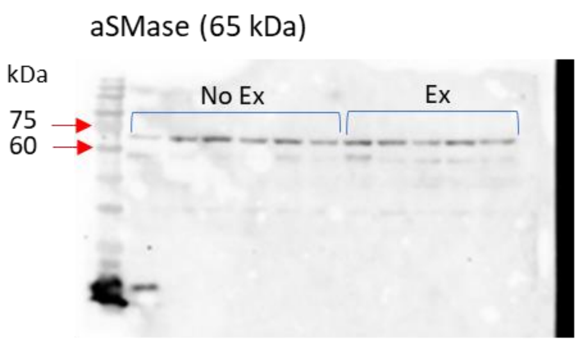

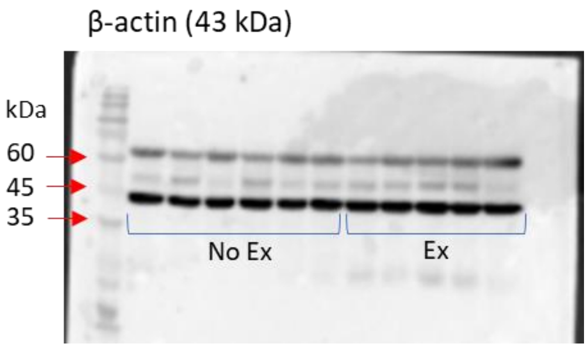

Absolute intensity

aSMase	1	2	3	4	5	5
No Ex	5731.669	8312.669	5510.426	4522.719	5247.841	7328.426
Ex	10716.13	7781.012	9152.134	8253.426	11802.55	
β-actin	1	2	3	4	5	5
No Ex	15840.79	15640.72	13053.55	14815.01	13295.6	12867.84
Ex	13255.13	14477.01	14399.01	15165.43	13720.43	

Internal normalization

aSMase/ β-actin	1	2	3	4	5	6
No Ex	0.36	0.53	0.42	0.31	0.39	0.57
Ex	0.81	0.54	0.64	0.54	0.86	
0.43	1	2	3	4	5	6
No Ex	0.84	1.23	0.98	0.71	0.92	1.32
Ex	1.88	1.25	1.48	1.26	2.00	

Fold change to control

	Aver.	SEM
No Ex	1.00	0.10
Ex	1.57	0.16

aCDase (α-subunit: 13 kDa)

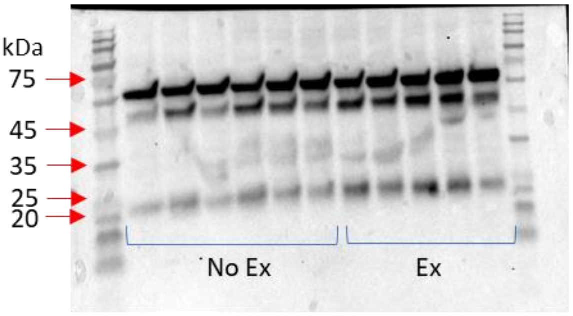

β-actin (43 kDa)

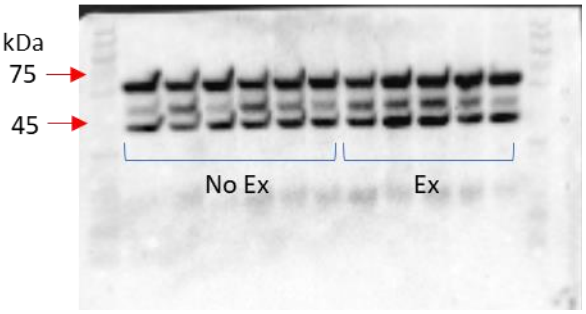

Absolute intensity

aCDase alpha subunit	1	2	3	4	5	6
No Ex	5525.669	9296.083	6087.305	11160.67	8934.255	8173.719
Ex	13455.38	10793.84	14284.26	12292.01	7556.305	

β-actin	1	2	3	4	5	6
No Ex	15840.79	15640.72	13053.55	14815.01	13295.6	12867.84
Ex	13255.13	14477.01	14399.01	15165.43	13720.43	

Internal normalization

aCDase alpha/β-actin	1	2	3	4	5	6
No Ex	0.35	0.59	0.47	0.75	0.67	0.64
Ex	1.02	0.75	0.99	0.81	0.55	

0.58	1	2	3	4	5	6
No Ex	0.60	1.03	0.81	1.30	1.16	1.10
Ex	1.76	1.29	1.72	1.40	0.95	

Fold change to No Ex

	Aver.	SEM
No Ex	1.00	0.12
Ex	1.42	0.15

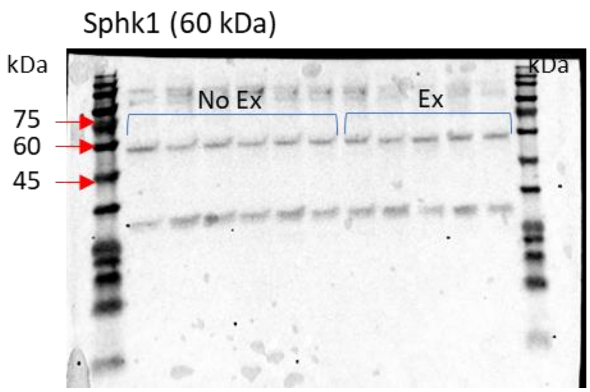

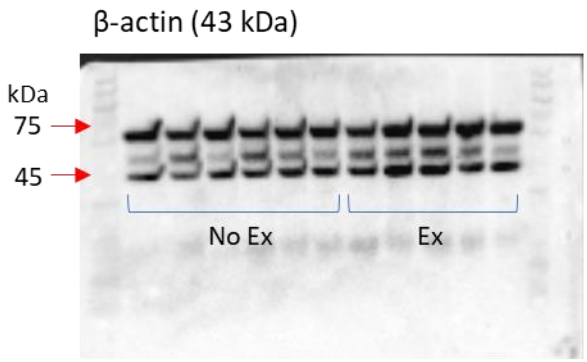

Absolute intensity

Sphk1	1	2	3	4	5	5
No Ex	17397.08	13627.38	10251.55	9822.426	8902.719	8115.426
Ex	8225.426	8774.426	14493.43	9893.719	7678.426	

β-actin	1	2	3	4	5	5
No Ex	15840.79	15640.72	13053.55	14815.01	13295.6	12867.84
Ex	13255.13	14477.01	14399.01	15165.43	13720.43	

Internal normalization

Sphk1/β-actin	1	2	3	4	5	6
No Ex	1.10	0.87	0.79	0.66	0.67	0.63
Ex	0.62	0.61	1.01	0.65	0.56	

0.79	1	2	3	4	5	6
No Ex	1.40	1.11	1.00	0.84	0.85	0.80
Ex	0.79	0.77	1.28	0.83	0.71	

Fold change to control

	Aver.	SEM
No Ex	1.00	0.09
Ex	0.88	0.10

Supplementary Figure S5. Uncropped blots.

1.2.1 Supplementary Table

Supplementary Table S1. Enriched canonical pathways in IPA (Attached in Excel file).

Supplementary Table S2. Absolute concentration of ceramide species (pmol/mg) in BP melanoma tumor homogenates. ***** $p<0.05$ vs No Ex. (Attached in Excel file).

Supplementary Table S3: Enriched canonical pathways in IPA. (Attached in Excel file).

Supplementary Table S4: Absolute concentration of ceramide species (pmol/mg) in BP melanoma tumors.

Ceramide species	No Ex (n=7)	Ex (n=8)
C14	6.7 ± 1.2	6.2 ± 0.9
dhC16	12.1 ± 1.3	8.9 ± 0.9*****
C16	307.9 ± 25.8	326.5 ± 12.3
C18	20.3 ± 3.0	14.8 ± 0.6
C18:1	1.8 ± 0.5	3.0 ± 0.6
C20	22.3 ± 2.9	16.0 ± 1.5
C20:1	0.4 ± 0.1	0.8 ± 0.2
C22	34.8 ± 3.0	38.1 ± 4.1
C22:1	11.1 ± 0.8	11.5 ± 1.3
C24	124.8 ± 7.0	127.9 ± 29.7
C24:1	520.8 ± 38.0	571.7 ± 29.7
C26	0.5 ± 0.1	0.5 ± 0.1
C26:1	4.4 ± 0.5	4.7 ± 0.4
Sum	1067.9 ± 70.2	1130.5 ± 51.6
